# Multi-omic analysis of longitudinal acute myeloid leukemia patient samples reveals potential prognostic markers linked to disease progression

**DOI:** 10.3389/fgene.2024.1442539

**Published:** 2024-09-27

**Authors:** Nisar Ahmed, Irene Cavattoni, William Villiers, Chiara Cugno, Sara Deola, Borbala Mifsud

**Affiliations:** ^1^ College of Health and Life Sciences, Genomics and Precision Medicine, Hamad Bin Khalifa University, Doha, Qatar; ^2^ Hematology and Bone Marrow Transplant Unit, Ospedale Centrale Bolzano, Bolzano, Italy; ^3^ Department of Medical and Molecular Genetics, King’s College London, London, United Kingdom; ^4^ Advanced Cell Therapy Core, Research, Sidra Medicine, Doha, Qatar; ^5^ William Harvey Research Institute, Queen Mary University London, London, United Kingdom

**Keywords:** AML relapse, multi-omic analyses, chromatin reorganization, epigenetic modifications, Omni-C, ATAC-seq, RNA-seq

## Abstract

Relapse remains a determinant of treatment failure and contributes significantly to mortality in acute myeloid leukemia (AML) patients. Despite efforts to understand AML progression and relapse mechanisms, findings on acquired gene mutations in relapse vary, suggesting inherent genetic heterogeneity and emphasizing the role of epigenetic modifications. We conducted a multi-omic analysis using Omni-C, ATAC-seq, and RNA-seq on longitudinal samples from two adult AML patients at diagnosis and relapse. Herein, we characterized genetic and epigenetic changes in AML progression to elucidate the underlying mechanisms of relapse. Differential interaction analysis showed significant 3D chromatin landscape reorganization between relapse and diagnosis samples. Comparing global open chromatin profiles revealed that relapse samples had significantly fewer accessible chromatin regions than diagnosis samples. In addition, we discovered that relapse-related upregulation was achieved either by forming new active enhancer contacts or by losing interactions with poised enhancers/potential silencers. Altogether, our study highlights the impact of genetic and epigenetic changes on AML progression, underlining the importance of multi-omic approaches in understanding disease relapse mechanisms and guiding potential therapeutic interventions.

## Introduction

Acute myeloid leukemia (AML) manifests as a complex disease marked by a multitude of genetic mutations and dysregulated gene expression profiles stemming from genetic and epigenetic alterations. These factors shape the trajectory of AML progression and confer resistance to therapeutic modalities. In general, AML occurs at any age, but it is the most prevalent form of acute leukemia in adults with a median age at diagnosis of 68 years and an estimated 20,380 diagnoses and 11,310 related deaths were projected for 2023 (Kishtagari and Levine, 2021; Siegel et al., 2023). In the past decade, extensive research has focused on AML heterogeneity at disease onset, leading to improved classification (Döhner et al., 2022) and novel treatment agents (DiNardo and Cortes, 2016; Stein et al., 2017; Stone et al., 2017) that considerably help to achieve complete remission in most patients, however, the 5-year overall survival (OS) rates are still only at around 28% (Howlader N, et al., 2021). It is mainly due to the high relapse rate, as 40%–60% of patients relapse within 3 years and fail to respond to conventional chemotherapy regimen (Bejanyan et al., 2015; Karlsson et al., 2017; Verma et al., 2010). Unfortunately, most individuals who relapse ultimately die from the disease (Schlenk et al., 2018). Prognosis in case of relapse is typically more unfavorable, especially when the recurrence occurs within a year after the initial remission (Rasche et al., 2021). Furthermore, relapse is a primary factor contributing to treatment failure (Döhner et al., 2015).

Recently, several investigations have leveraged next-generation sequencing (NGS) to identify gene mutations specific to relapse in certain AML subgroups, aiming to elucidate the disease’s course. However, there is a considerable genetic heterogeneity among different relapsed patients (Jan et al., 2012; Metzeler et al., 2016; Papaemmanuil et al., 2016), even when focusing on specific AML subgroups (Ahn et al., 2018; Gröschel et al., 2015; Wang et al., 2016). This diversity hinders the discovery of consistent relapse-specific signatures. Additionally, there are only a few studies that compare the mutational profiles of diagnosis and relapse samples in AML patients, and they focus on coding mutations (Farrar et al., 2016; Masetti et al., 2016), despite growing evidence that non-coding mutations in regulatory elements or structural variants altering enhancer usage can also drive oncogenesis (Northcott et al., 2014; Zhu et al., 2020). This underscores the significance of epigenetic changes in the course of the disease and emphasizes the necessity of considering the intrinsic and extrinsic disease heterogeneity (Levin et al., 2021; Schwenger and Steidl, 2021; Vicente-Dueñas et al., 2018). Furthermore, there is a paucity of comprehensive molecular characterization of longitudinal AML samples, including diagnosis and relapse pairs. Therefore, we aim to assess the contribution of epigenetic factors, such as active regulatory elements and their long-range chromatin interactions, in conjunction with gene expression profiles across longitudinal samples, elucidating their role in the progression of AML.

To achieve this, we have profiled the long-range chromatin interactions (using Omni-C), open chromatin regions (using ATAC-seq), and gene expression (using RNA-seq) of two matched adult AML patients at diagnosis and relapse. We found significant alterations in the 3D chromatin landscape and accessible chromatin regions between relapse and diagnosis samples. Additionally, upregulated genes in relapse showed enrichment for H3K27me3 in distal regions of diagnosis-specific interactions, indicating loss of potential silencer connections during relapse.

## Methods

### Patient samples

We received live-frozen mononuclear cells from peripheral blood of adult AML diagnosis and relapse paired samples from Bolzano General Hospital, Italy. Each sample contained >50% blast cells Supplementary Table S1. The project was reviewed by the Hamad Bin Khalifa University Institutional Review Board and approved under protocol #QBRI-IRB 2020-02–017.

### Omni-C library preparation

We used the Omni-C kit (Dovetail Genomics), which is a sequence-independent endonuclease-based proximity-ligation protocol. Briefly, 1 × 10^6^ live bone marrow cells were fixed in DSG (disuccinimidyl glutarate), a non-cleavable and membrane-permeable protein-protein crosslinker, followed by formaldehyde to reversibly crosslink *in vivo* DNA-protein interactions. The fixed cells were treated with DNase I to digest chromatin. Next, for the proximity ligation, the chromatin ends were polished, and biotin-tagged bridges were used to create chimeric molecules. The crosslink of lysate was reversed, and the purified DNA was used for NGS library preparation. Finally, the library was enriched for ligation-containing chimeric molecules. The Omni-C libraries were sequenced on an average of 14x coverage on the Illumina HiSeq X Ten system with 151-base paired-end reads (>300M reads).

### ATAC-seq library preparation

One of our objectives was to interrogate active chromatin regions through an assessment of chromatin accessibility, which was evaluated using ATAC-seq, a method known for its capability to delineate regions of open chromatin. The Active Motif ATAC-seq kit was used to perform ATAC-seq on living cells in accordance with the Omni-ATAC-seq protocol described by (Corces et al., 2017). For ATAC-seq, cryopreserved bone marrow samples were slowly thawed using IMDM supplemented with 10% FBS and DNase. Viability was calculated under a hemocytometer with trypan blue–samples with a viability <80% were subjected to dead cell sorting using MACS dead cell removal kit (cat: 130-090–101). 1 × 10^5^ cells were taken forward for ATAC-seq.

Briefly, for sample preparation, 1 × 10^5^ cells were first pelleted and washed with ice-cold PBS. Subsequently, the cells were re-suspended in an ice-cold ATAC-Lysis buffer. Next, for tagmentation the isolated nuclei were incubated at 37°C for precisely 30 min while being shaken at 800 rpm in a transposition mixture containing 100 nM final transposase. For DNA purification Zymo DNA Clean and Concentrator-5 Kit was used. Following that, PCR amplifications of tagmented libraries were performed using 10 cycles of PCR and DNA was extracted using 60 µL SPRI beads for size selection. To assess size distribution, PCR-amplified libraries were analyzed with Bioanalyzer. Finally, libraries were sequenced on the Illumina HiSeq 4,000 platform to ∼50 million paired end 100bp reads.

### RNA-seq library preparation

Total RNA was purified from 5 × 10^5^ live bone marrow cells using a Qiagen RNeasy Plus Micro kit. Briefly, the cells were disrupted and homogenized using RLT buffer. Genomic DNA eliminator spin columns were used to remove the DNA, and RNeasy MinElute spin columns were used to purify RNA. Total RNA-seq libraries were prepared using TruSeq Stranded Total RNA Library Prep Gold Kit. The prepared libraries were sequenced using the Illumina Nextseq platform to ∼60–50 million paired-end 101bp reads.

### Omni-C data pre-processing

Omni-C libraries were processed through an in-house pipeline. The quality control (QC) of sequencing of Omni-C libraries was performed on fastq files using FastQC (Andrews et al., 2010). The adaptor sequences from the reads were trimmed using TrimGalore. The trimmed read pairs were aligned using BWA-MEM (version 0.7.17 or higher) to the GRCh38 version of the human genome. To find ligation junctions in Omni-C libraries, the “pairtools parse” module was used by setting MAPQ greater or equal to 40 and walks-policy as 5unique. The pairtools pipeline records the strand of each paired read and the outermost (5′) aligned base pair into a “pairsam” file upon identification of a ligation event in the alignment file. The pairsam format records Hi-C pair information along with SAM entries, which was then sorted using “pairtools sort”. The “pairtools dedup” command was used to remove PCR duplicates from sorted pairsam files and here we also produced the statistics of the library by using the flag “–output-stats”. Finally, the deduplicated pairsam files were then used to create two different files, such as pairs file and BAM file, which can be used for further downstream processing.

### Omni-C library QC and complexity

To check the quality of Omni-C libraries, we have used the stats files calculated by “pairtools dedup”, which contains information on total reads, mapped reads, duplicate reads, and total read pairs. In addition, we used the get_qc.py pipeline from Dovetail Genomics to summarize these stats in percentage and absolute values. We have checked the complexity of Omni-C libraries using the lc_extrap utility of the preseq package from Smith lab (github.com/smithlabcode/preseq), which aims to predict the complexity of sequencing libraries.

### Contact matrix

The contact maps, which are compressed and sparse formats, are produced from the pairs files using Juicer tools (Durand et al., 2016). The pairs files were first converted into HiC files, which are highly compressed binary representations of the contact matrices using “pre” command of Juicer tools. The HiC contact matrices were finally visualized using Juicebox (J. T. Robinson et al., 2018).

### Differential interaction analysis

The systematic biases such as those arising from enzyme digestion, DNA ligation, and PCR amplification from Omni-C libraries were corrected using the HiCorr pipeline (Lu et al., 2020). To prepare the input, the BAM files were sorted according to co-ordinates and read pairs were then mapped to select the cis and trans read pairs. The HiCorr outputs were then used to apply a deep learning-based tool called DeepLoop (Zhang et al., 2022) to perform loop signal enhancement. We have used the pre-built models from DeepLoop to improve the sensitivity, robustness, and quantitation of Omni-C loops and output chromatin loop strength.

After that, we have created a count matrix (*M*_(i, j)_) where each row *(i)* represents a chromatin loop and column *(j)* represents a sample and it was populated with the loop strengths from DeepLoop. The count matrix was normalized using the R Bioconductor package edgeR (M. D. Robinson et al., 2010). The normalized counts were used to calculate the standard deviation, and we selected the top 100,000 most variable interactions based on standard deviation. Finally, the matrix with only the most variable interactions was used to perform differential interaction analysis using the R Bioconductor package limma (Ritchie et al., 2015).

### Omni-C downstream analysis

Exploratory analysis was performed in R version 4.3.1 using the Bioconductor package GenomicRanges (Lawrence et al., 2013). All interaction landscapes were visualized in the WashU Epigenome Browser (Li et al., 2022).

### ATAC-seq library pre-processing

Briefly, all libraries underwent FastQC testing (Andrews et al., 2010) to evaluate the library quality and make sure that each library is free of significant problems like low read quality or adapter contamination. Next, we used Trimmomatic (Bolger et al., 2014) with default parameters to filter low-quality reads, and Truseq adaptors were trimmed off from the reads. Subsequently, these reads were aligned to the hg38 version of the human genome using Bowtie2 aligner, which created SAM files that were converted to BAM files using samtools. After that, mitochondrial, duplicate, and blacklisted reads were removed using samtools and bedtools. Reads were shifted to adjust for tn5 binding using the alignmentSieve tool. Finally, peaks were then called on the final processed BAM files using the callpeak command with BAMPE of the MACS2 peak calling algorithm.

### ATAC-seq downstream analysis

MACS2 peaks were further used for downstream processing. Peaks were assigned to genomic elements using BioMart (Durinck et al., 2005) and TxDb.Hsapiens.UCSC.hg38. knownGene R Bioconductor packages (Carlson, et al., 2015). All exploratory analysis was performed in R version 4.3.1 using the GenomicRanges Bioconductor package (Lawrence et al., 2013).

### RNA-sequencing pre-processing

The QC of RNA-seq libraries was performed on fastq files using the FastQC tool (Andrews et al., 2010). The adaptor sequences from the reads were trimmed using TrimGalore. The trimmed reads were aligned to the hg38 genome using STAR and RSEM was used to calculate the expression values as expected counts from the aligned RNA-seq data. The count matrix was normalized using the R Bioconductor package edgeR (M. D. Robinson et al., 2010). Finally, the normalized counts were used to analyze differential gene expression using the R Bioconductor package limma (Ritchie et al., 2015). Significant upregulated and downregulated genes with adjusted *p*-value <0.1 were selected based on log2 fold change >1 and log2 fold change < −1, respectively.

### RNA-seq downstream analysis

The normalized reads were used for all exploratory analysis and plots were generated using custom code in R version 4.3.1. Volcano plots were created using the R Bioconductor package ggplot2.

### Multi-omic data integration

Significant differential Omni-C interactions were used to identify relapse-specific and diagnosis-specific anchors in each sample and subsequently, we specified diagnosis-specific ATAC-seq peaks by taking the overlap of two diagnosis samples and relapse-specific ATAC-seq peaks by taking the overlap of two relapse samples. Further, we removed the common peaks between diagnosis-specific and relapse-specific ATAC-seq peaks. Next, relapse-specific and diagnosis-specific Omni-C anchors and ATAC-seq peaks were overlapped using the Bioconductor package GenomicRanges (Lawrence et al., 2013) to investigate which differentially interacting regions are accessible. Finally, we assigned genes to these regions using BioMart (Durinck et al., 2005).

Furthermore, we investigated which differentially expressed genes are linked to differentially accessible DNA in differentially interacting regions (20 Kb). Then, we calculated enrichment for differentially expressed genes in relapse and diagnosis-specific interactions in comparison with 10 random, size-matched sets of genes from the whole genome. Finally, the distal or other end of the interactions of upregulated genes that have diagnosis-specific Omni-C interactions and ATAC-seq peaks were overlapped with publicly available H3K27me3 ChIP-seq data (accession number GSM4565992) from CD34^+^ common myeloid progenitor cells, to test whether their upregulation can be attributed to the loss of a silencer contact. For these analyses, the R Bioconductor package GenomicRanges (Lawrence et al., 2013) was used.

### Motif enrichment

For the differential enrichment of transcription factor binding motifs within differentially accessible regions found in differentially interacting regions the Homer (Hypergeometric Optimization of Motif EnRichment) motif suite of tools was used (Heinz et al., 2010). The parameters findMotifsGenome.pl with -size −200,200 to centre peaks to a 400bp region and -bg to set background of peaks. Motifs with FDR<0.01 were considered significantly enriched. Relapse-specific anchors and peaks were used as target sequences, and diagnosis-specific anchors and peaks were used as background sequences.

## Results

### Overview of multi-omics assays in diagnosis and relapse AML

**FIGURE 1:**
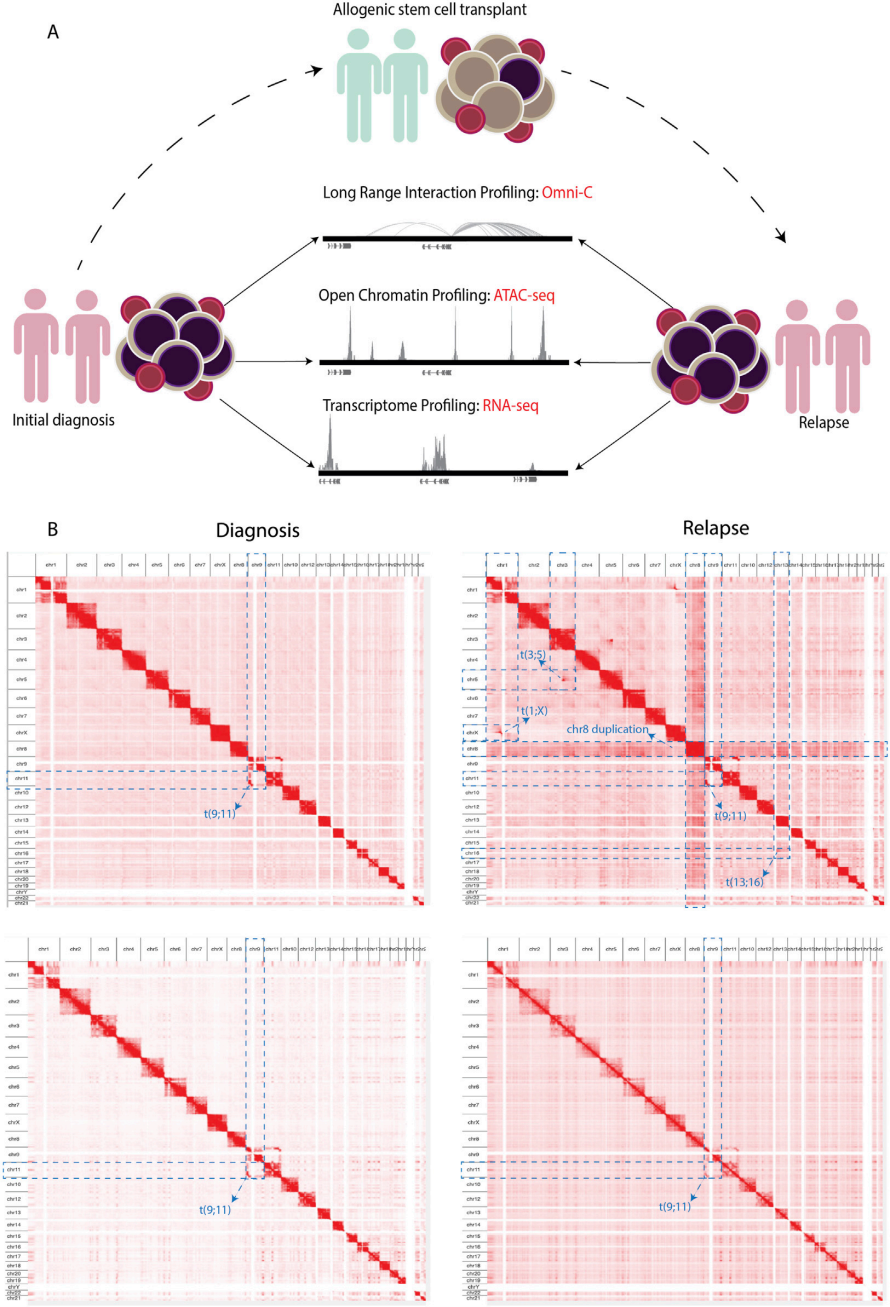
**(A)** Schematics showing the experimental workflow where PBMCs obtained from adult AML samples at initial diagnosis underwent NGS library preparation for Omni-C, ATAC-seq, and RNA-seq analyses. Subsequently, the same NGS libraries were prepared from samples upon relapse following allogenic stem cell transplantation. **(B)** Contact maps derived from the Omni-C dataset, depicting the chromosomal architecture of diagnosis samples (*left*) compared with relapse samples (*right*). The blue dotted lines show the chromosomal abnormalities each sample contains genome wide.

We integrated changes of the 3D genome structure, chromatin accessibility, and gene expression to decipher the molecular changes that occur in AML at relapse compared to at the time of diagnosis in two initial diagnoses and relapse AML sample pairs (Supplementary Table S1). We explored long-range regulatory interaction patterns using Omni-C, active regulatory elements using ATAC-seq, and transcriptional patterns through RNA-seq (Figure 1A). In addition, we used Omni-C data to create genome-wide contact maps and assess the spectrum of large chromosomal changes in these AML samples. The contact maps identified that all of the samples had a t(9; 11) or *KMT2A::MLLT3* translocation. This confirmed the initial diagnosis of one patient; however, this translocation was undetected by cytogenetics in the other patient. The KMT2A::MLLT3 subtype of AML is shown to have a poor/intermediate prognosis, and the current mechanistic understanding of KMT2A-rearrangement (KMT2Ar) prognosis has not fully translated into therapeutic success due to the complexity of genomic events contributing to the disease (Krivtsov and Armstrong, 2007; Liedtke and Cleary, 2009; Meyer et al., 2018). We additionally observed within our Omni-C data that relapse samples had gained extra chromosomal abnormalities, for example, chromosome eight duplication, t(3; 5), and t(1; X) (Figure 1B; Supplementary Figure S1A–D). Such abnormalities are common occurrences at relapse in AML (Kern et al., 2002).

### Differential interactions and differentially accessible regions in relapse *versus* diagnosis samples

**FIGURE 2:**
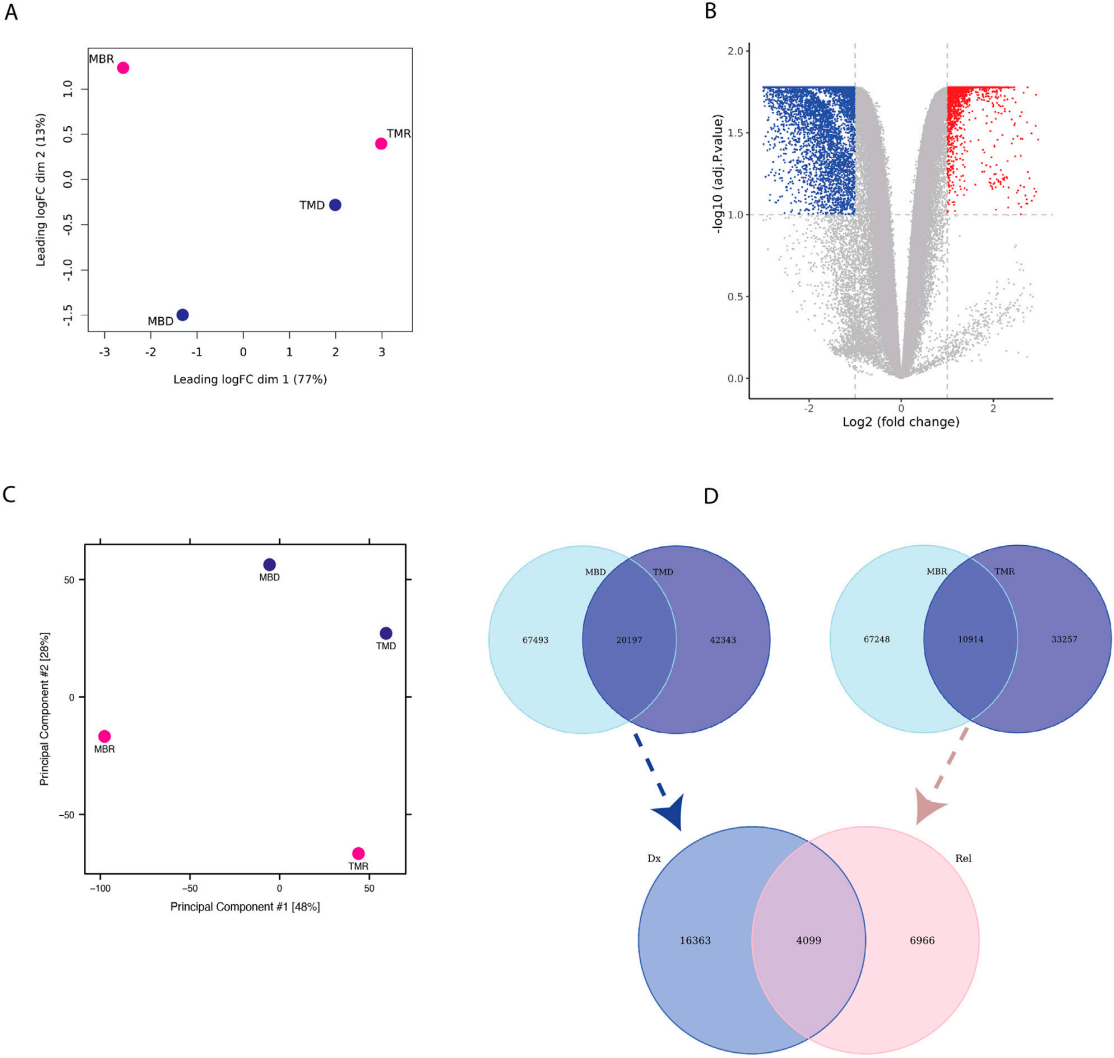
**(A)** PCA plot based on limma logfold change (logFC) of Omni-C data **(B)** Volcano-plot showing differential interaction of relapse *versus* diagnosis interactions based on Omni-C datasets where each dot represents an interaction. Blue represents significant diagnosis-specific interactions (5,262), and red represents significant relapse-specific interactions (2,940), whereas grey dots represent non-significantly differential interactions. **(C)** PCA plot based on ATAC-seq normalized peak intensity. **(D)** Venn diagram representing the overlap of diagnosis-specific ATAC-seq peaks (*upper left*), relapse-specific ATAC-seq peaks (*upper right*) between patients and of consistent diagnosis-specific and relapse-specific ATAC-seq peaks (*bottom*).

Next, we asked whether there are any regions that are differentially interacting in the genome distinguishing relapse from diagnosis samples. While we found higher intra-patient similarity than intra-status (Figure 2A), we could detect common diagnosis and relapse-specific interactions. By comparing relapse *versus* diagnosis samples, we found 8,202 significantly differential interactions (p-adj <0.1), where 5,262 interactions were defined as diagnosis-specific, and 2,940 interactions were found to be relapse-specific (Figure 2B). This highlights distinctive interaction patterns between diagnosis and relapse timepoints that are consistent across patients. Next, we compared the chromatin accessibility profile of the diagnosis and relapse pairs using ATAC-seq. We estimated the similarity between relapse and diagnosis samples using PCA based on the global open chromatin profile, which again showed higher inter-patient variability than inter-state variability (Figure 2C). We performed an overlap analysis between two diagnosis samples and two relapse samples, where we took the common diagnosis (20,197) and common relapse (10,914) open chromatin regions. Subsequently, by overlapping common diagnoses and relapse peaks, we identified 16,363 consistent diagnosis-specific open chromatin regions and 6,966 relapse-specific regions in addition to 4,099 regions that were active in all samples (Figure 2D).

### Integration of differential chromatin interaction and accessibility signatures

**FIGURE 3:**
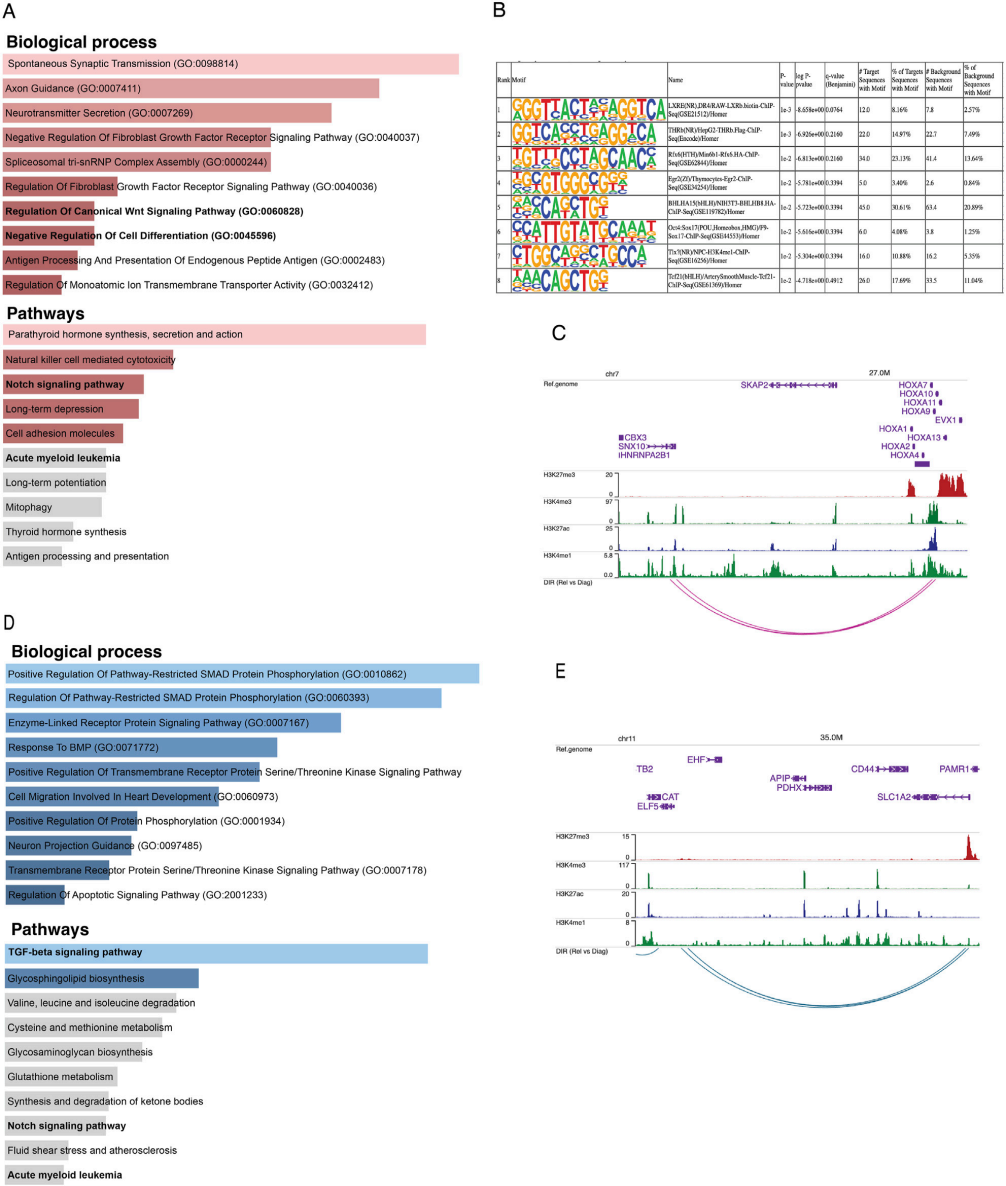
**(A)** Bar plots representing the enrichment of biological processes and pathways based on genes annotated to consistent relapse-specific interactions and peaks. **(B)** The top eight motifs enriched in relapse-specific Omni-C anchors and ATAC-seq peaks compared to diagnosis-specific anchors and peaks. **(C)** Example of a relapse-specific Omni-C interaction and ATAC-seq peaks where the *HOXA9* promoter region interacts with a distal active enhancer at the downstream region of the *SNX10* gene. **(D)** Bar plots representing the enrichment of biological processes and pathways based on genes annotated to diagnosis-specific interactions and peaks. **(E)** Example of a diagnosis-specific interaction and ATAC-seq peaks at the promoter region of *ELF5* interacting with a poised enhancer within *SLC1A2*. Arcs represent long-range chromatin interactions, active enhancers are marked by H3K27ac and H3K4me1, and poised enhancers are marked by H3K27me3 and H3K4me1.

We integrated the Omni-C anchors with ATAC-seq peaks to investigate which differential interacting regions are also differentially accessible (Supplementary Figure S2A). We found 150 unique relapse-specific anchors that were differentially accessible in relapse samples, which were annotated with 107 genes. GO-term analysis of these genes reveals the biological processes and pathways in which these genes were enriched, such as regulation of canonical Wnt signaling pathway, negative regulation of cell differentiation, Notch signaling pathway, and AML (Figure 3A). Next, we investigated if relapse-specific peaks in relapse-specific anchors are associated with different transcription factors (TFs) compared to diagnosis-specific ones by performing differential motif discovery on these two sets. We noted that a liver X receptor beta (LXRb) was enriched in relapse-specific regions (Figure 3B). Finally, we explored whether these differential interactions are anchored at active or poised enhancers. For instance, *HOXA9*, which is involved in cell differentiation and upon dysregulation, contributes to leukemogenesis, was found to form a relapse-specific long-range interaction with an active enhancer at the 3′UTR of the *SNX10* gene, marked by H3K4me1/3 and H3K27ac (Figure 3C). In addition, we found KMT2Ar subtype-specific patterns at known KMT2A target genes, such as *UBE2J1* and *PARP8*, in the loop anchors (Supplementary Figures S2B, S2C). Overexpression of these genes contributes to the blockage of normal hematopoietic differentiation and promotes leukemogenesis.

On the other hand, we found 312 unique diagnosis-specific anchors that were differentially accessible and in proximity to these peaks, 154 genes were present. Further, these genes were enriched in biological pathways such as TGF-beta and Notch signaling (Figure 3D). Examples of genes with diagnosis-specific interactions include *ELF5*, which is associated with epithelial cell function and has implications in cancer. Its promoter formed a diagnosis-specific contact with a poised enhancer marked by H3K27me3 and H3K4me1, likely contributing to its low expression in diagnosis samples (Figure 3E). Additionally, a TGF-beta pathway member, *BMP8A*, also interacted with a poised enhancer. This gene plays critical roles in various cellular processes, such as tissue differentiation, cell proliferation, and apoptosis. *ST8SIA1*, which is involved in cell signaling, recognition, and adhesion, was interacting with an active enhancer near the 3′UTR of *CMAS*. Another gene with a diagnosis-specific active enhancer contact is *HPSE2*, which has a role in tumor microenvironment dynamics and cancer progression. Its promoter is interacting with an active enhancer present in the proximity of its own 3′UTR Supplementary Figure S2D–F).

### Analysis of differential gene expression dynamics

**FIGURE 4:**
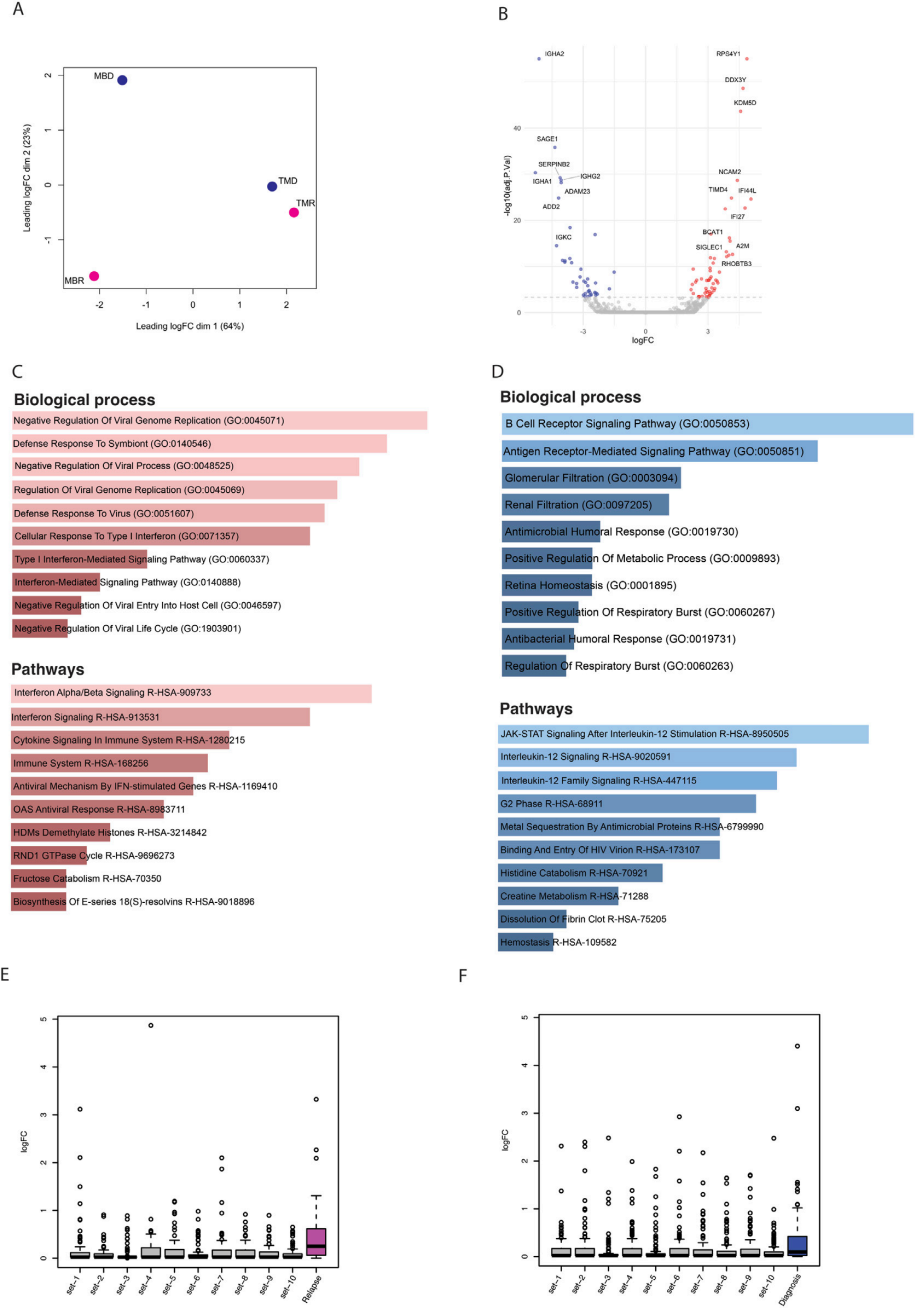
**(A)** PCA plot based on logfold change (logFC) of gene expression level. **(B)** Volcano plot showing significantly (p-adj <0.1) differentially expressed genes; here blue dots are significantly downregulated genes, red dots are significantly upregulated, and grey are nonsignificant genes. **(C)** Bar plots showing the enrichment of biological processes and pathways based on upregulated genes. **(D)** Bar plots showing enrichment of biological processes and pathways based on downregulated genes. **(E)** Box plots representing the logfold change of upregulated genes that overlapped with relapse-specific interactions and peaks (pink) and of 10 different random sets of upregulated genes. **(F)** Box plots representing the logfold change of upregulated genes that overlapped with diagnosis-specific interactions and peaks (blue) and of 10 different random sets of upregulated genes.

Finally, we explored the gene expression profile of these longitudinal samples using RNA-seq. Similarly to what we found using the other omics methods, samples do not cluster together based on disease status, i.e., relapse and diagnosis (Figure 4A). Nonetheless, we asked whether there are differentially expressed genes between relapse *versus* diagnosis samples. Using limma-voom, we found overall, 95 significantly dysregulated genes that were selected using p. adj <0.1 and absolute log2FC > 1 (Figure 4B). These genes were enriched in pathways like B-cell receptor signaling pathway and immune and defense mechanism-related biological processes, as well as AML (Supplementary Figure S2G). Further, out of 95 significant dysregulated genes identified by comparing relapse with diagnosis, there were 55 upregulated genes (Supplementary Table S2) and 40 downregulated genes (Supplementary Table S3). These upregulated genes were enriched in defense response and interferon and cytokines signaling (Figure 4C), whereas downregulated genes were enriched in B-cell receptor, Jak-STAT, and interleukin signaling pathways (Figure 4D).

### Multi-omic data integration exploration

Finally, we integrated all three omic datasets together to identify consistently differentially regulated genes across relapse and diagnostic timepoints. While there were only a handful of genes that showed significant differences, we noted that in general, genes that were associated with relapse-specific interactions and open chromatin regions showed higher levels of upregulation than those genes associated with diagnosis-specific interactions and open chromatin regions, as well as random genes from the genome, indicating that relapse-specific interactions are often new active enhancer contacts (Figures 4E, F). Some of the genes that were associated with diagnosis specific interactions and open chromatin regions were also upregulated in relapse samples. We hypothesized that these upregulated genes may have initially interacted with silencers/poised enhancers, but upon relapse, this interaction is lost (Supplementary Figure S3A). We found 13 genes (*ST8SIA1*, *IVD*, *ACAN*, *KIF5C*, *ADAMTS5*, *CFAP299*, *PITX2*, *KLHL31*, *RBPMS*, *SNAI2*, *SOX17*, *CSMD3*, and *DMRTA1*) where the diagnosis-specific interaction linked them to regions marked by H3K27me3, which is a significantly higher proportion than expected by chance (*p*-value = 9.99 × 10^−5^) (Supplementary Figures S3B, C). In contrast, genes associated with either relapse-specific or diagnosis-specific interactions and open chromatin regions showed a similar level of downregulation compared to random genes from the genome (Supplementary Figures S3D, E). In an independent study, we found that out of these 13 genes, nine genes lost chromatin accessibility upon relapse (Nuno et al., 2024) (Supplementary Figure S4).

There were five genes that showed significant differences in all three analyses. Our findings revealed that among genes that were upregulated in relapse, *SDC2* and *CD70* were in consistent relapse-specific anchors and peaks, while *NCAM2* and *IFI44* were in consistent diagnosis-specific anchors and peaks. These genes have been shown to have some link with different types of cancers. For example, the SDC2 (syndecan-2) protein functions as an integral membrane protein and participates in cell proliferation, cell migration and cell-matrix interactions via its receptor for extracellular matrix proteins, and altered *SDC2* expression has been detected in several different tumor types (Akl et al., 2015; Canarte et al., 2023). Among the downregulated genes in relapse, *TSPYL5*, which is a *TP53* suppressor via its interaction with USP7 (Epping et al., 2011), was found in a consistent diagnosis-specific anchor and peak. Dysregulation of most of these genes was corroborated by independent studies. Using nine diagnosis and relapse pair samples, Nuno et al. found that *NCAM2*, *IFI44,* and *TSPYL5* significantly lost their accessibility upon relapse (Supplementary Figure S5). Finally, explored the relationship between the expression of these genes and clinical outcome, in terms of survival, using the Leucegene AML RNA-seq prognostic cohort (n = 373). We observed that high expression of these genes, including *TSPYL5*, negatively impacts the overall survival of AML patients, except for *CD70*, which did not show a significant effect (Supplementary Figure S6).

## Discussion

AML aggressive subtypes are often linked to rearrangements in the mixed lineage leukemia gene (KMT2Ar). 10% of adult acute leukemias with a very poor prognosis and chemoresistance are caused by clinically significant and genetically well-defined KMT2Ar (Krivtsov and Armstrong, 2007; Liedtke and Cleary, 2009; Meyer et al., 2018). Therefore, it is important to understand what genetic and epigenetic changes characterize relapse. Patients with t(9; 11)(p22; q23), the most frequent translocation which leads to the *KMT2A::MLLT3* fusion gene*,* carried by the patients in this study, show relatively acceptable results with intensive chemotherapy (Grimwade et al., 2010; Mrózek et al., 1997; Stölzel et al., 2016; Chen et al., 2013; Pigneux et al., 2015), placing them in the intermediate risk group according to ELN 2017 and ELN 2022 classifications (Döhner et al., 2017; 2022). This highlights the importance of identifying the oncogenic translocation for clinical decision making. Our chromatin conformation data revealed an undetected 9;11 translocation in one patient, indicating the need for more in-depth karyotyping using next-generation sequencing-based techniques. One option is targeted RNA-seq, which is becoming routine in diagnostics to detect low level fusion genes (Kerbs et al., 2022).

Advances in pharmacological inhibitors and targeted immuno-therapies have considerably improved the treatment options for KMT2Ar leukemias (Issa et al., 2023; van der Sluis et al., 2023). However, KMT2Ar leukemias show highly heterogenous response to therapeutic regimens despite their similar oncogenic lesions (Issa et al., 2023; Lambo et al., 2023). Common genetic mechanisms leading to therapeutic resistance include clonal selection and the acquisition of secondary mutations. Additionally, a subset of these leukemias may also evade targeted therapies through epigenetic mechanisms that remain poorly understood (Tirtakusuma et al., 2022).

In this study, we explored epigenetic and transcriptomic changes in AML progression by integrating Omni-C, ATAC-seq, and RNA-seq data in two pairs of diagnosis and relapse samples. We found substantial chromatin remodeling, which was indicated by differences in the 3D chromatin structure, with a significant loss of chromatin interactions and open chromatin regions in relapse. Furthermore, differential gene expression analysis of those longitudinal samples revealed that upregulated genes are enriched in broad immune response pathways, including those related to cytokine signaling, especially interferon-alpha/beta signaling, while downregulated genes show enrichment in signaling pathways associated with Jak-STAT and interleukin.

Combined multi-omics data pinpointed three upregulated genes (*SDC2, NCAM2,* and *IFI44*) that showed negative effects on survival in a larger leukemia cohort. *TSPYL5* was downregulated in both of the relapse samples we tested, and higher expression of this gene was also associated with worse prognosis in the Leucegene cohort. In conclusion, our integrated analysis highlights distinct genomic and epigenomic profiles in relapse compared to diagnosis. Although the small sample size limits our study, the genes highlighted here showed dysregulation and had a prognostic effect in independent patient cohorts warranting their further exploration in larger cohorts to assess their clinical relevance and the therapeutic implications of these observations for KMT2Ar AML.

## Data Availability

The datasets presented in this study can be found in online repositories. The names of the repository/repositories and accession number(s) can be found below: https://www.ncbi.nlm.nih.gov/geo/, GSE267375 https://www.ncbi.nlm.nih.gov/geo/, GSE267376.

## Supplementary material

SUPPLEMENTARY FIGURE S1 Each bar plot represents the average read coverage of cis interactions across chromosomes. **(A, B)** show the average coverage at diagnosis **(C, D)** show the average coverage at relapse. **(C)** is the relapse sample of **(A)**, **(D)** is the relapse sample of **(B)**.

SUPPLEMENTARY FIGURE S2 
**(A)** Schematics of identifying relapse-specific differential interactions and peaks and diagnosis-specific interactions and peaks. **(B-F)** Examples of relapse-specific **(B, C)** and diagnosis-specific **(D–F)** Omni-C interactions and ATAC-seq peaks of UBE2J1 and PARP8; and BMP8A, ST8SIA1, and HPSE2, respectively. Red track shows H3K27me3, blue shows H3K27ac, and green shows H3K4me3/1 in Common Myeloid Progenitor – CD34 positive cells. **(G)** Bar plots showing the enrichment of biological processes and pathways based on significantly dysregulated genes between relapse versus diagnosis.

SUPPLEMENTARY FIGURE S3
**(A)** Schematics portraying the interactions of promoter regions with distal poised enhancers marked by H3K27me3. **(B)** Examples of gene promoters interacting with silencers. **(C)** Bar plots showing the proportion of diagnosis-specific and random interactions where the distal element is marked by H3K27me3. Error bars indicate the standard deviation of the random proportions based on 10,000 Monte Carlo simulations. **(D)** Box plots representing downregulated genes that overlapped with relapse-specific interactions and peaks and their comparison with the expression level of 10 different random sets of downregulated genes.**(D)** Box plots representing downregulated genes that overlapped with diagnosis-specific interactions and peaks and their comparison with the expression level of 10 different random sets of downregulated genes.

SUPPLEMENTARY FIGURE S4 Box plots representing ATAC-seq data from Nuno et al. of 18 stable initial diagnosis (blue) and relapse (brown) AML samples. 13 genes, which were upregulated and lost their interaction with a silencer. Diagnosis and relapse sample pairs are connected by grey lines. The log2FC differences and adjusted p-values are taken from the differential accessibility analysis between relapse and diagnosis using gene score,(a metric to estimate the accessibility of chromatin regions associated with specific genes).

SUPPLEMENTARY FIGURE S5 Box plots representing ATAC-seq data from Nuno et al. of 18 stable initial diagnosis (blue) and relapse (brown) AML samples. These five genes were found consistent across all three analyses. Diagnosis and relapse sample pairs are connected by grey lines. The log2FC differences and adjusted p-values were taken from the differential accessibility analysis between relapse and diagnosis using gene score, a metric to estimate the accessibility of chromatin regions associated with specific genes.

SUPPLEMENTARY FIGURE S6 Kaplan-Meier plots showing the overall survival (OS) of patients from the Leucegene Acute Myeloid Leukemia (AML) cohort based on gene expression levels of five different genes that we found significant. Each displays the survival probability over time, stratified by gene expression levels (above or below median). Patients with above-median gene expression levels of **(A)** SDC2, **(B)** CD70, **(C)** NCAM2, **(D)** IFI44, and **(E)** TSPYL5 show significant differences in survival compared to those with below-median levels, with p-values of 0.0004, 0.0194, 0.0001, 0.0135, and 0.0003, respectively.

SUPPLEMENTARY TABLE S1 Clinical characteristics of AML patients at diagnosis and relapse.

SUPPLEMENTARY TABLE S2 List of upregulated genes of relapse versus diagnosis AML samples with logFC >1 and adj-pval < 0.1.

SUPPLEMENTARY TABLE S3 List of downregulated genes of relapse versus diagnosis AML samples with logFC < −1 and adj-pval < 0.1.
