# Supplementary figures and images for: Multi-omic analysis of longitudinal acute myeloid leukemia patient samples reveals potential prognostic markers linked to disease progression
Source: Front Genet. 2024 Sep 27;15:1442539. doi: 10.3389/fgene.2024.1442539 (PMC11466779; doi:10.3389/fgene.2024.1442539)

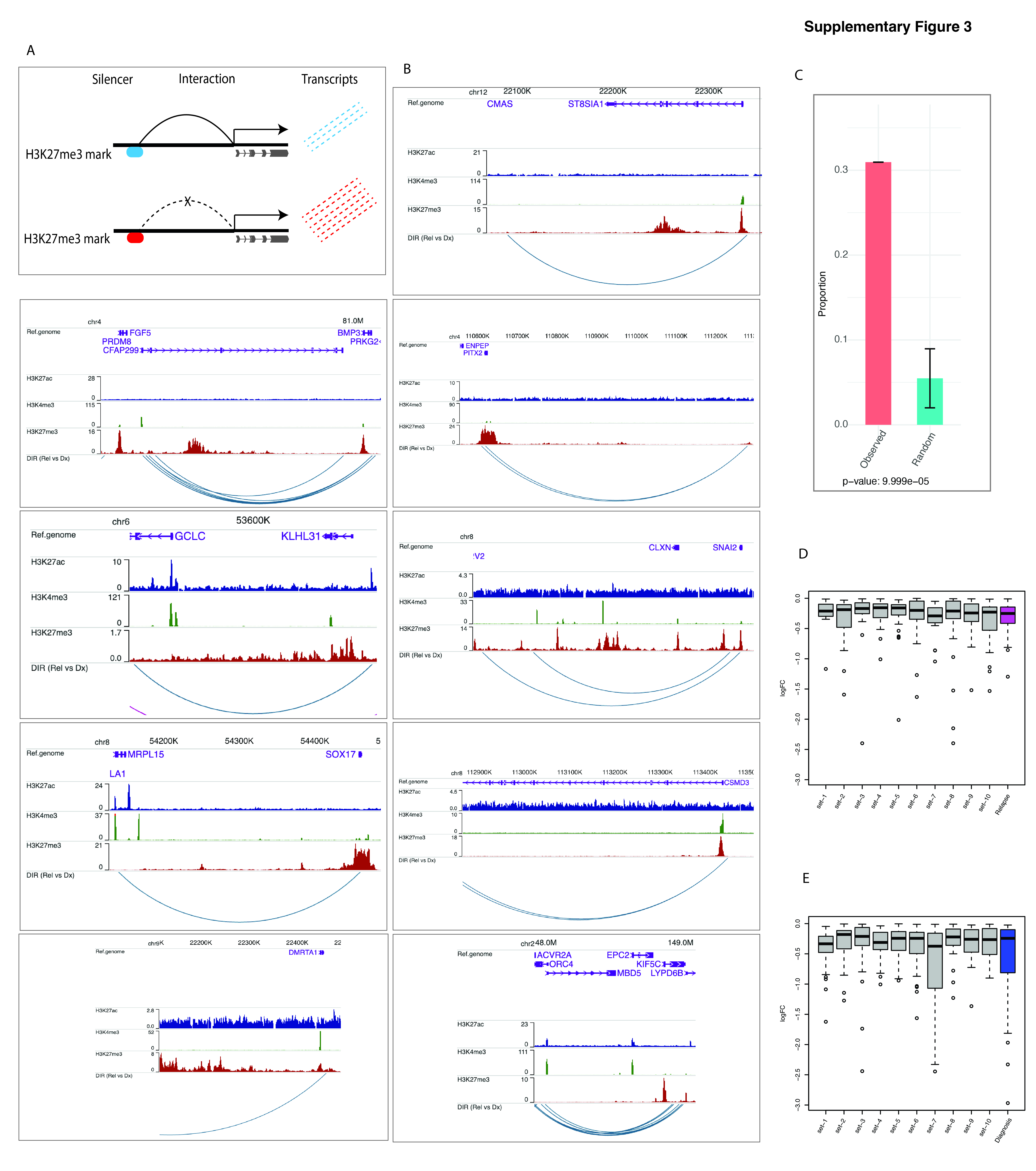

Supplement: Supplementary file 2 [file Image3.JPEG]

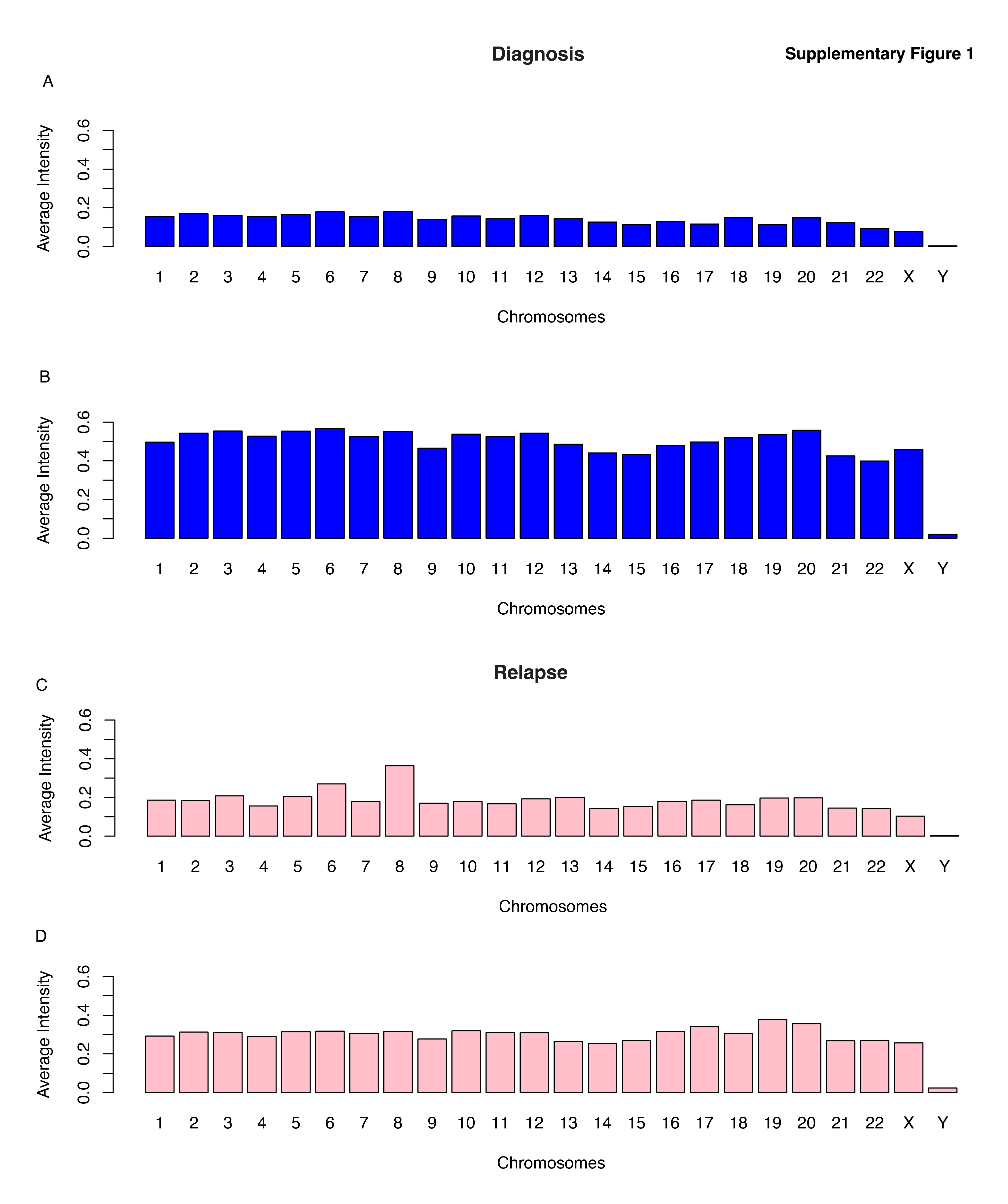

Supplement: Supplementary file 4 [file Image1.JPEG]

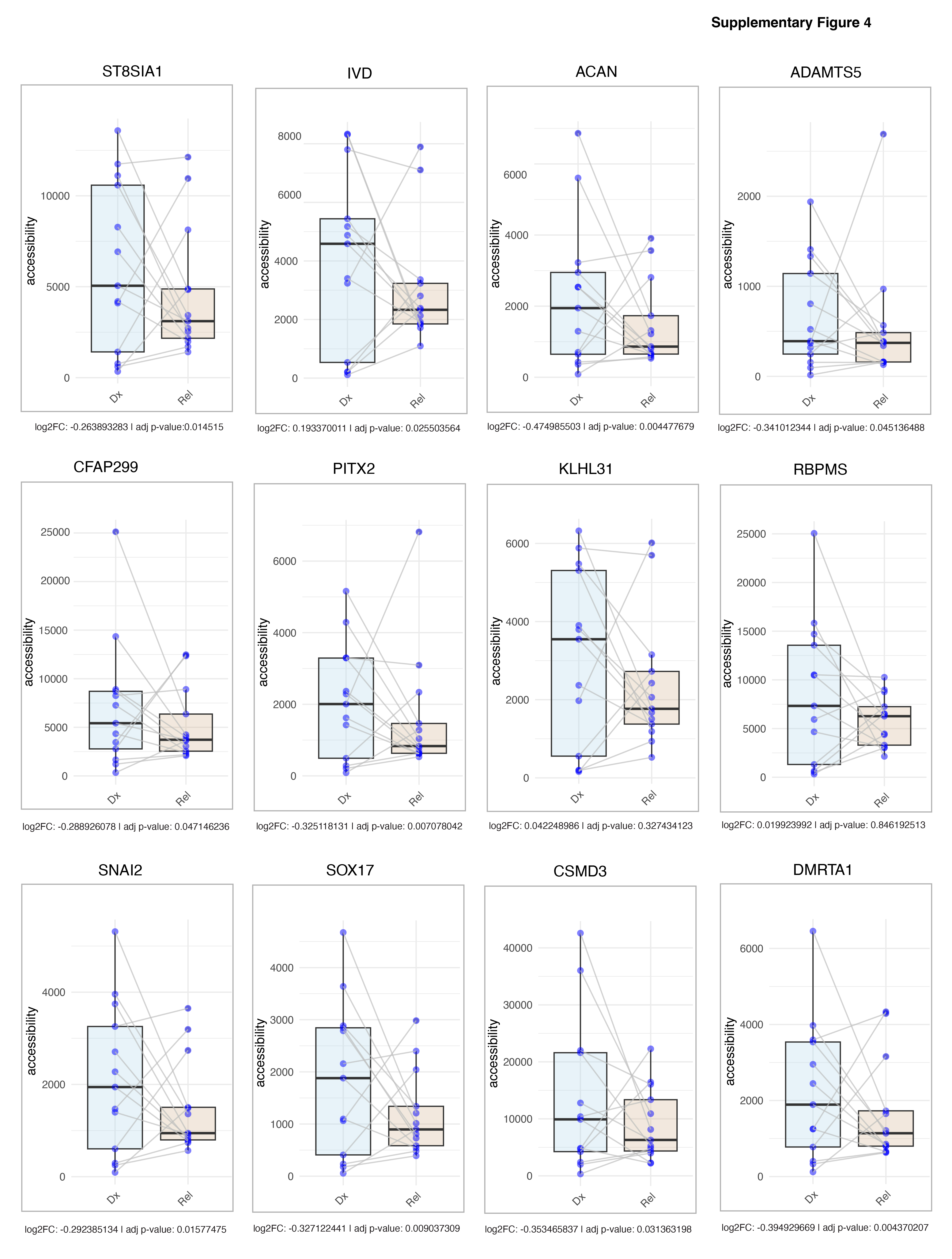

Supplement: Supplementary file 5 [file Image4.JPEG]

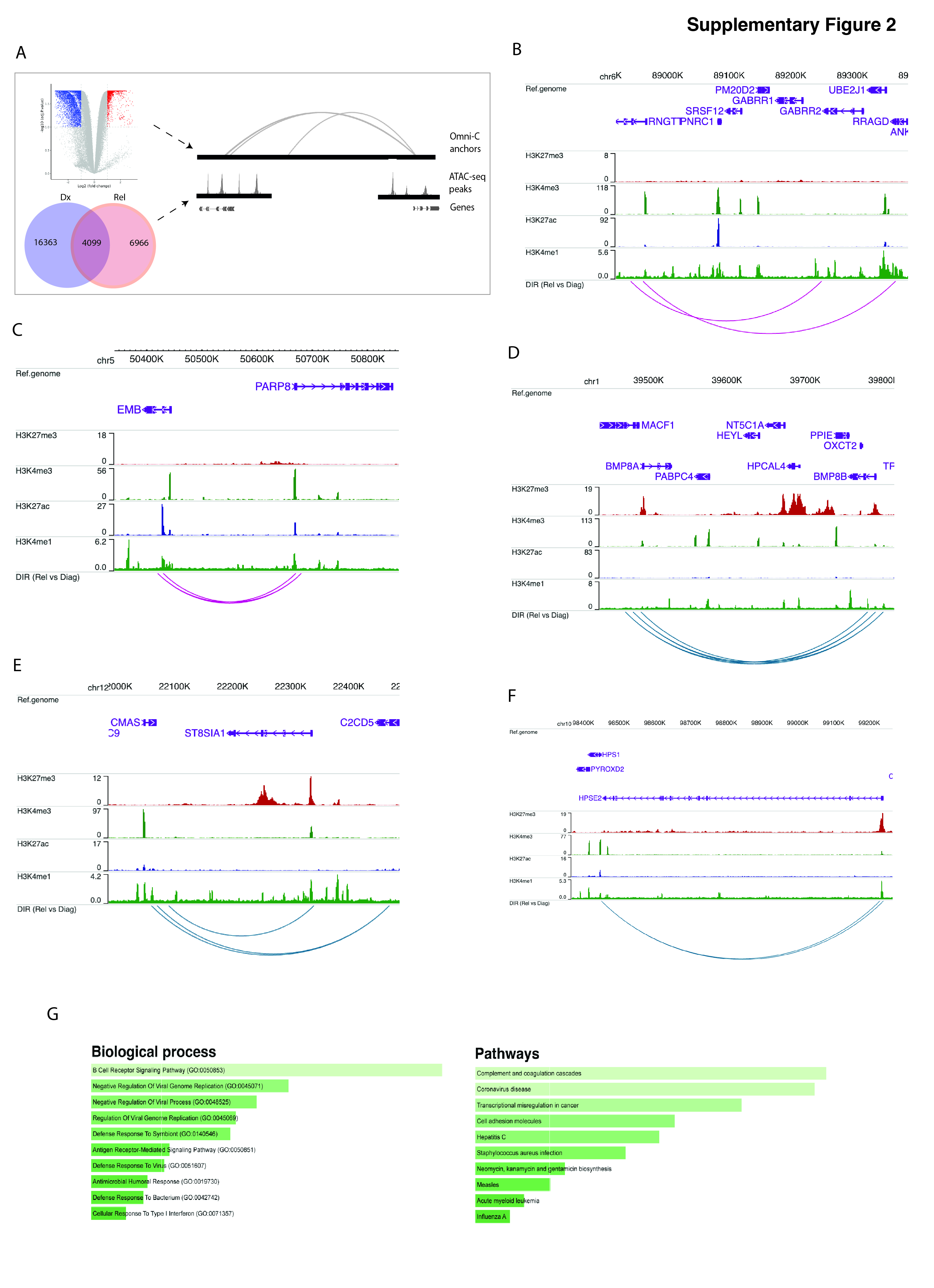

Supplement: Supplementary file 6 [file Image2.JPEG]

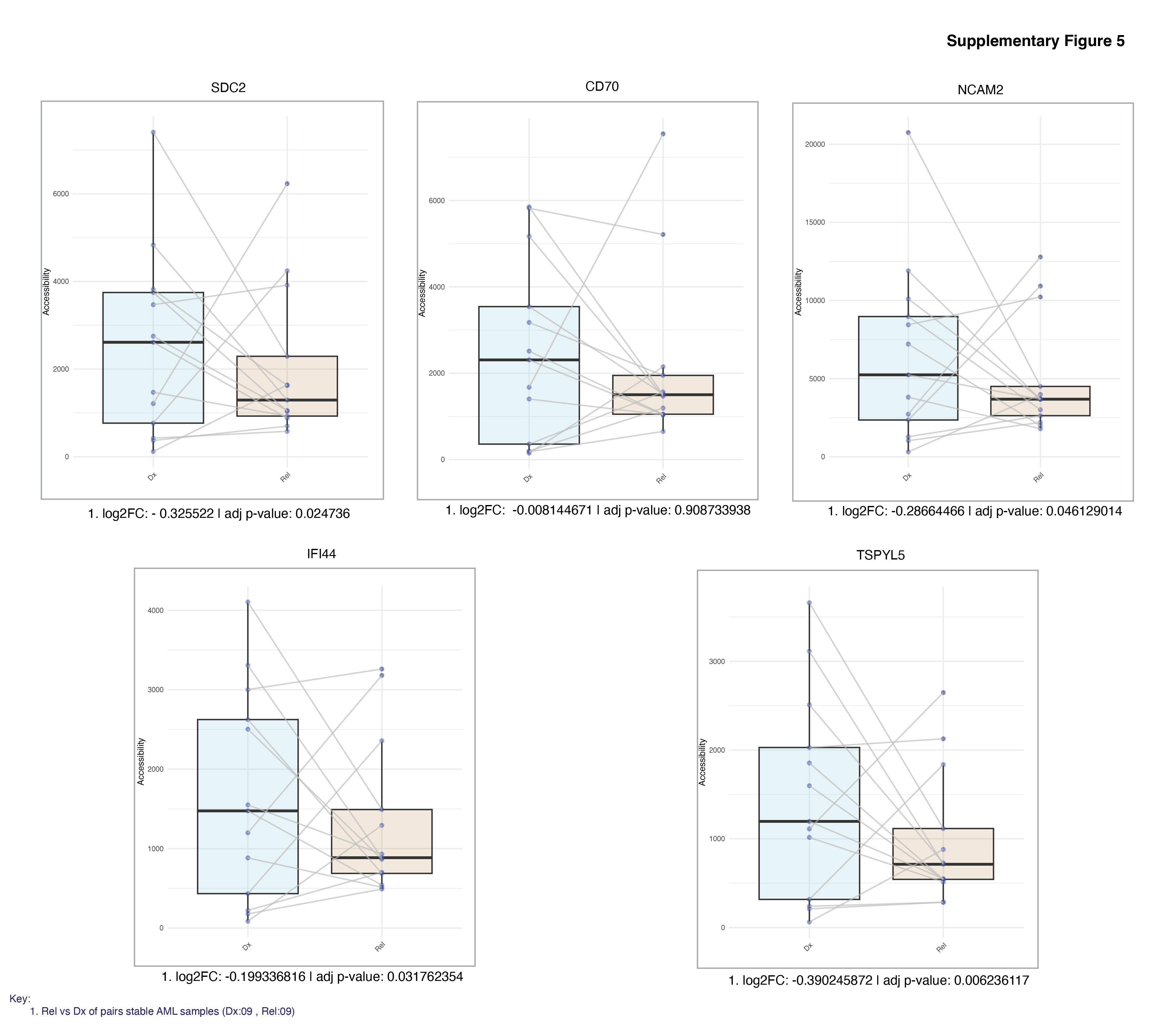

Supplement: Supplementary file 7 [file Image5.JPEG]

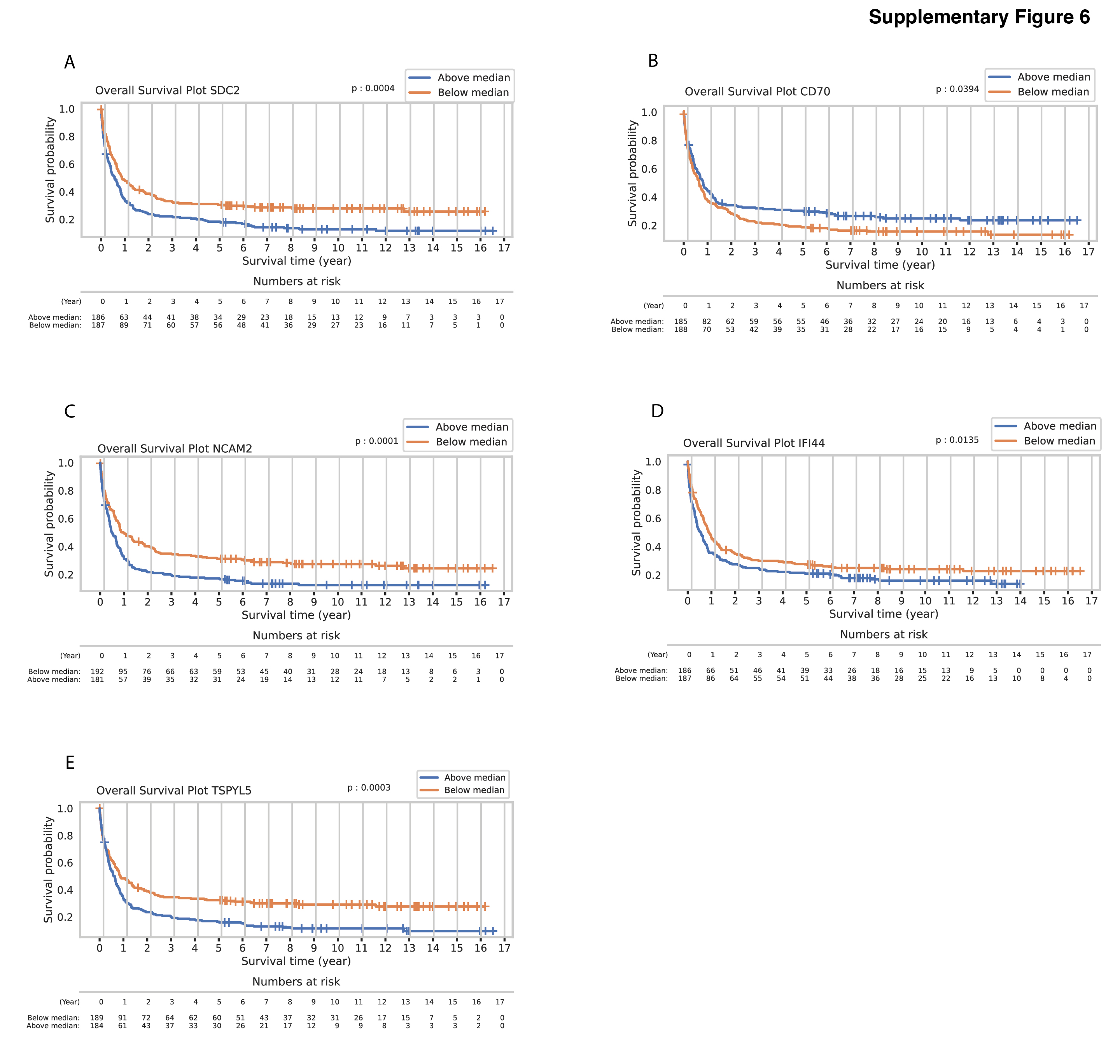

Supplement: Supplementary file 9 [file Image6.JPEG]
